# Progression and mortality of patients with cystic fibrosis in China

**DOI:** 10.1186/s13023-024-03522-1

**Published:** 2025-01-07

**Authors:** Wangji Zhou, Yaqi Wang, Yanli Yang, Yanyan Sun, Chongsheng Cheng, Jinrong Dai, Shuzhen Meng, Keqi Chen, Yang Zhao, Xueqi Liu, Dingding Zhang, Song Liu, Weiguo Zhu, Yaping Liu, Kai-Feng Xu, Xinlun Tian

**Affiliations:** 1 Department of Pulmonary and Critical Care Medicine, State Key Laboratory of Complex Severe and Rare Diseases, State Key Laboratory of Common Mechanism Research for Maior Diseases, Peking Union Medical College Hospital, Chinese Academy of Medical Sciences and Peking Union Medical College, Beijing, China; 2 Center of Medical Research, Peking Union Medical College Hospital, Chinese Academy of Medical Sciences, Peking Union Medical College, Beijing, China; 3 Center for Bioinformatics, National Infrastructures for Translational Medicine, Institute of Clinical Medicine & Peking Union Medical College Hospital, Chinese Academy of Medical Sciences and Peking Union Medical College, Beijing, China; 4 Department of Primary Care and Family Medicine, State Key Laboratory of Complex Severe and Rare Diseases, Peking Union Medical College Hospital, Chinese Academy of Medical Sciences and Peking Union Medical College, Beijing, China; 5 The State Key Laboratory for Complex, Severe, and Rare Diseases, The State Key Sci-tech Infrastructure for Translational Medicine, Peking Union Medical College Hospital, Beijing, China

**Keywords:** Chinese, Cystic fibrosis, Lung function, Prognosis, Mortality

## Abstract

**Background:**

Patients with cystic fibrosis (CF) are rare in China and differ significantly from the Caucasian populations in terms of clinical and genetic characteristics. However, the progression and mortality of Chinese patients with CF have not been well described.

**Results:**

This study included all 67 patients from the Peking Union Medical College Hospital CF cohort, with a median followed up time of 5.2 years. Compared to patients diagnosed with CF in childhood, adult-diagnosed patients exhibit a lower proportion of pancreatic exocrine insufficiency (25.0% vs. 77.8%, *P* = 0.001) and a higher body mass index (19.6 vs. 17.7 kg/m^2^, *P* = 0.045). According to the mixed-effects model, for patients ≤ 30 years of age at diagnosis, FEV_1_% predicted decreased 1.17% per year. The generalized linear regression model showed that higher baseline FEV_1_% predicted and occurrence of pulmonary exacerbations were associated with the progression of patients with CF. The survival rates at 5 years and 10 years after the diagnosis were 96.7% and 80.6%, respectively. The log-rank test showed baseline FEV_1_% predicted < 50%, and high CF-ABLE and 3-year prognostic scores were associated with mortality in patients with CF in China.

**Conclusions:**

We reported the progression and mortality of patients with CF in China, which was a rare and relatively unknown population in the past. Baseline FEV_1_% predicted is associated with progression and mortality. Pulmonary exacerbations can accelerate the decline in lung function. The CF-ABLE and 3-year prognostic scores are applicable for predicting poor prognosis in patients with CF in China.

**Supplementary Information:**

The online version contains supplementary material available at 10.1186/s13023-024-03522-1.

## Background

Cystic fibrosis (CF) is one of the most common autosomal recessive diseases in Caucasians, with an incidence of approximately 1 in every 3,500–5,000 newborns [1]. The disease is caused by mutations in the cystic fibrosis transmembrane conductance regulator (*CFTR*) gene, which mainly affects the function of chloride-conducting transmembrane channel, thereby causing bronchiectasis, pancreatic exocrine insufficiency (PI), male infertility, and elevated sweat chloride levels [2].

However, CF is quite rare in China, with about only 200 patients reported thus far [2]. Our previous study found that Chinese patients with CF primarily have pulmonary symptoms with fewer digestive symptoms. Mutation p. Gly970Asp (G970D) is the most common variant in Chinese patients, while p. Phe508del (F508del), the most common mutation in Caucasians, is rare in Chinese people [3–7]. Therefore, we believe that Chinese patients with CF are significantly different from Caucasian patients in clinical and genetic characteristics.

Pulmonary insufficiency is the major cause of progression and mortality of patients with CF. Percent predicted forced expiratory volume in one second (FEV_1_% predicted) is regarded as the best available measure for assessing CF [8]. Studies in Caucasian populations have shown that body mass index (BMI), chronic *Pseudomonas aeruginosa* (PA) infection, and PI are associated with decreased FEV_1_% predicted [9], and underweight and multiple exacerbation patients are at increased risk of death [10]. A series of prediction tools such as CF-ABLE score and 3-year prognostic score are available to predict death/lung transplantation in Caucasian patients with CF [11, 12]. Adult-diagnosed patients with CF are a special subset of the CF population, with milder phenotype and genotype. Previous studies have suggested that the rate of decline of FEV_1_% predicted in these patients is slower than that of patients diagnosed in childhood [13, 14].

Chinese patients with CF were a rare and relatively unknown population in the past. And contemporary studies on the progression and mortality of patients with CF in China are lacking. There is only one study regarding the prognosis of Chinese pediatric patients, and the lung function of 17 patients showed no significant changes during a median follow-up of 2.8 years [15]. The Peking Union Medical College Hospital (PUMCH) CF cohort established a database of patients with CF in China with both pediatric- and adult-diagnosed patients in a regular follow-up plan, which is the longest duration of follow-up cohort of Chinese patients with CF reported currently. In this study, we aimed to investigate the progression and mortality of Chinese patients with CF and analyzed the determinant factors, to improve the understanding of these patients as a distinct group from Caucasians.

## Methods

### Patient cohort

The cohort was extracted from the CF Registry Study at PUMCH between January 2012 and October 2023 and all patients met the diagnosis criteria according to the 2017 consensus guidelines for CF diagnosis from the Cystic Fibrosis Foundation (e-Table 1). According to the guidelines, CF diagnosis should be made if a patient has relevant clinical features or a positive family history with one of the following: (1) Sweat chloride value ≥ 60 mmol/L; (2) Sweat chloride value in the intermediate range (30–59 mmol/L) and occurrence of 2 CF-causing *CFTR* mutations [16]. The patients were followed up regularly in our hospital and evaluated annually during routine visits. At the time of evaluation, the patients were in a stable condition with no pulmonary exacerbations (PEx) within 4 weeks.

**Fig. 1:**
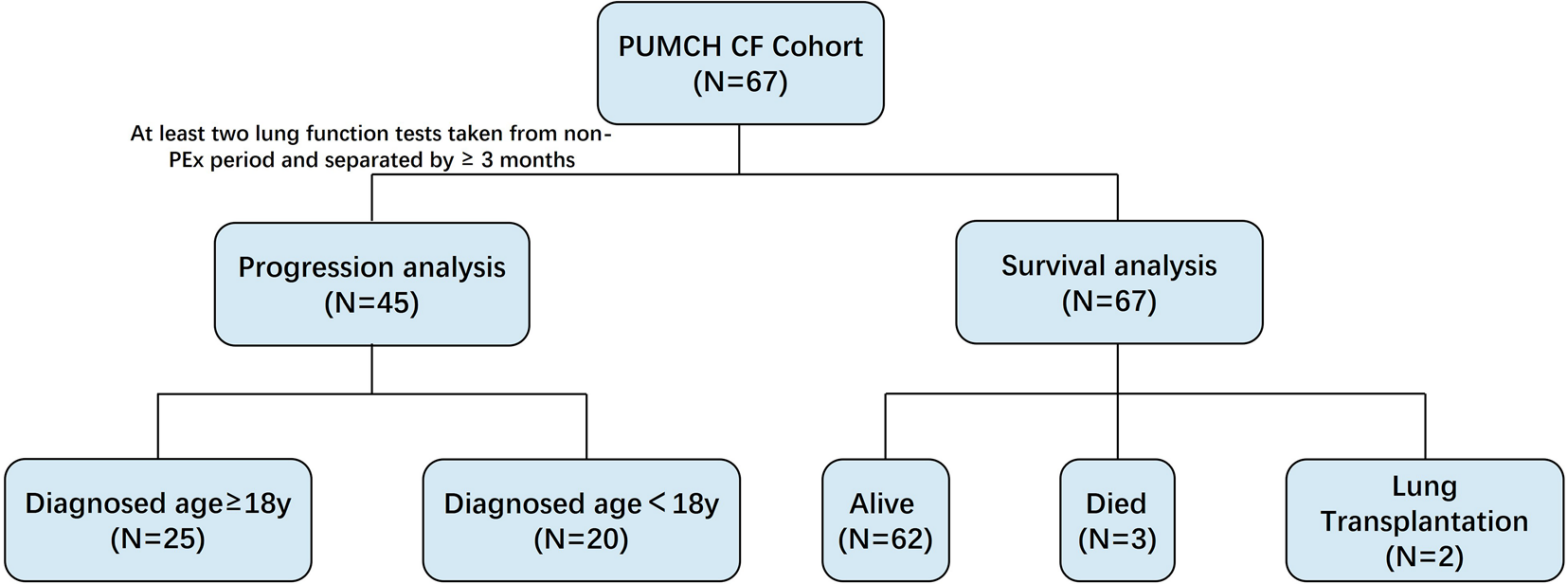
Flow chart showing the cohort analysis. CF = cystic fibrosis; PEx = pulmonary exacerbation; PUMCH = Peking Union Medical College Hospital

The inclusion criteria were as follows: (1) a definitive diagnosis of CF and (2) baseline evaluation. Patients with incomplete baseline evaluations (Refer to “Study Variables” for details) were excluded. For patients who have at least two lung function tests taken from non-PEx period and separated by ≥ 3 months, their lung function results are used to progression analysis of CF (Fig. 1).Informed consent was obtained from all patients or their legal guardians. The study was approved by the Ethics Committee of PUMCH (Identifier: JS-2639) and was carried out in accordance with the tenets of the Declaration of Helsinki.

### Study variables

We collected the following information: (1) age at diagnosis, sex, birthplace, symptoms at diagnosis (pulmonary, gastrointestinal, pulmonary and gastrointestinal, or male infertility); (2) *CFTR* mutations classified as the presence or absence of G970D; (3) PI (based on fecal fat microscopy by Sudan III); (4) other clinical variables at baseline (defined as within 2 years after the diagnosis date) were also included, such as lung function, BMI, PExs requiring hospitalization, sputum culture results, presence of allergic bronchopulmonary aspergillosis (ABPA) along with CF, CF-ABLE score, and 3-year prognostic score. The outcome of lung function of interest, FEV_1_% predicted, was obtained before the bronchodilator test. All lung function data were obtained from patients prior to lung transplantation. Male infertility was defined according to the following criteria: (1) absence of sperm after semen centrifugation or (2) reproduction through assisted reproductive technology after marriage.

### Survival follow-up

The follow-up started from the diagnosis of CF and ended at death, on lung transplantation, when lost to follow-up, or October 31, 2023, whichever occurred first. Patients were followed up via regular clinic visits, telephone calls, text messages, and the patient follow-up platform, Xingshulin MedClip app (Xingshulin Information Technology [Beijing] Limited Company, Beijing, China).

### Statistical analyses

Continuous variables are reported as mean ± standard deviation (SD) or median (interquartile range [IQR]), and categorical variables are described as the proportional percentage, No. (%). We used the *Student*’s t test or the Wilcoxon rank-sum test for continuous variables and the chi-square test for categorical variables.

For progression, a linear mixed-effects model was used to describe the trajectory of FEV_1_% predicted by age from diagnosis to last visit, lung transplantation, or death. In the mixed-effects model, age at diagnosis and individual variation were random effects and individual-specific trajectories were treated as random slopes. To better fit the trajectory of FEV_1_% predicted with age, the models of quadratic and cubic relationship between FEV_1_% predicted and age were also constructed, and more details of the models are shown in e-Table 2. The best model was chosen based on the Akaike information criterion and Bayesian information criterion. Due to the small number of patients diagnosed over the age of 30, we excluded these patients for sensitivity analysis to evaluate the stability of the model (e-Table 3). The best model was chosen based on the Akaike information criterion and Bayesian information criterion. As only five patients were older than 30 years of age at diagnosis, we adopted the mixed effect model of sensitivity analysis in the subsequent analysis to better evaluate the factors related to the change of lung function. Univariate and multivariable generalized linear regression models were used to further investigate the factors related to changes in FEV_1_% predicted. The rate of decline in FEV_1_% predicted was calculated as the change between FEV_1_% predicted at last visit (which was predicted by overall mixed-effects model) and baseline FEV_1_% predicted divided by the time gap between the two values.

Kaplan–Meier curves were drawn to describe survival, and the log-rank test was used for comparisons between groups. Because of the small number of patients who died or underwent lung transplantation, the multivariable Cox proportional hazards model was not applied.

Statistical tests were two-sided, and statistical significance was set at *P* < 0.05. All data were analyzed using the R version 4.3.2 software (R Foundation for Statistical Computing, Vienna, Austria).

## Results

### Baseline characteristics of patients from the PUMCH CF cohort

**Fig. 2:**
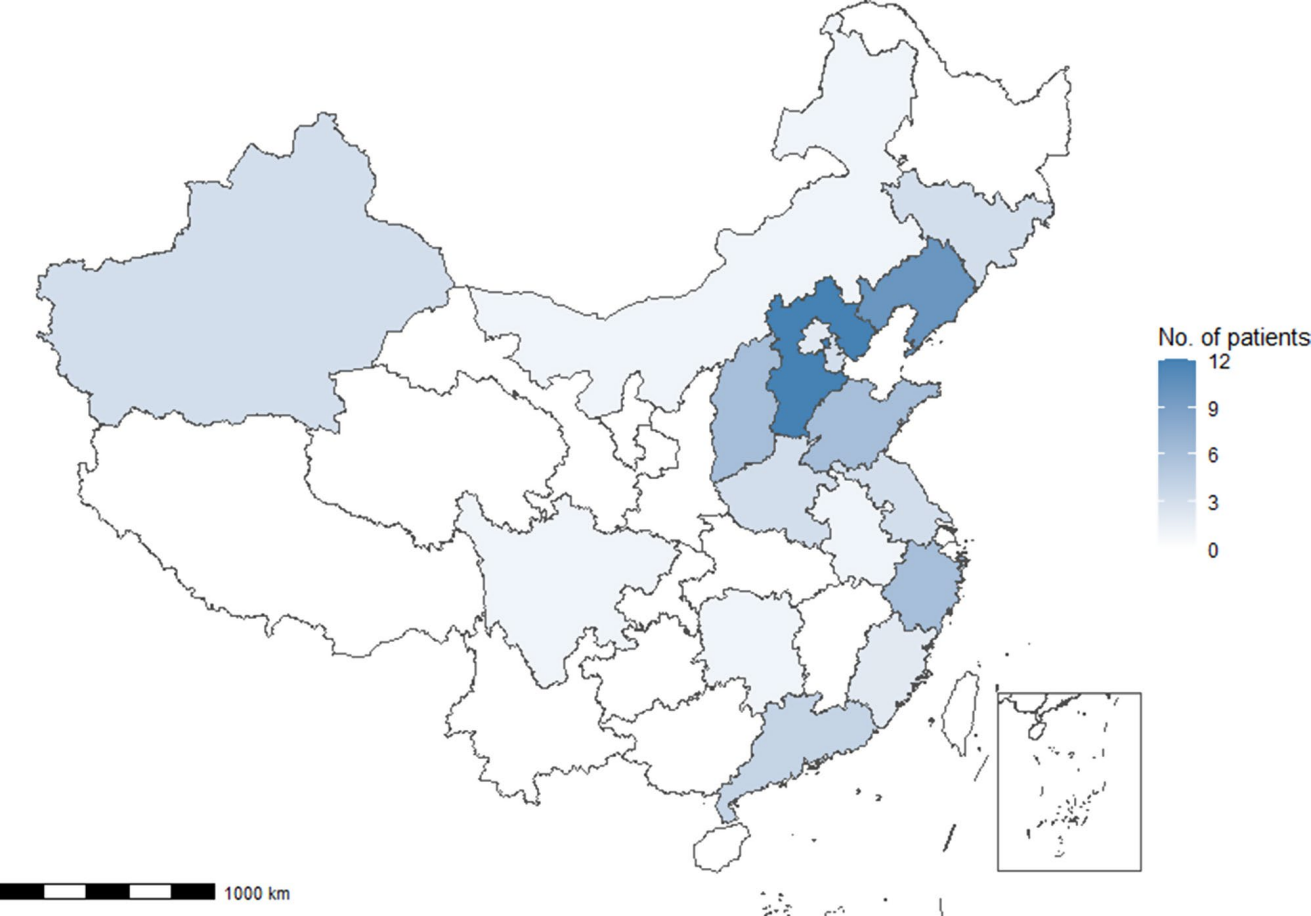
Graph showing the distribution of the birthplace of each patient in the PUMCH CF cohort. CF = cystic fibrosis; PUMCH = Peking Union Medical College Hospital

This study included all 67 patients from the PUMCH CF cohort from 17 provinces, municipalities, and autonomous regions in China (Fig. 2). Patients were followed up for a median of 5.2 years; three patients died and two patients received lung transplantation. The average age at diagnosis was 17.5 years, with slightly higher number of males than females. Nearly half of the patients (46.3%) were diagnosed during adulthood. Almost all patients had pulmonary symptoms at diagnosis and 28.2% also had gastrointestinal symptoms. Male infertility was also common (92.9%). Twenty-four patients (36.9%) had *CFTR* G970D mutation, of which three were homozygous. During follow-up, 43.1% of patients had PI. The baseline characteristics of the patients are presented in Table 1, and they were divided into two groups based on whether they were > 18 years old at diagnosis. Compared with adult-diagnosed patients, patients diagnosed at childhood had a higher proportion of PI and a lower BMI.

**Table 1:** Baseline characteristics of all 67 patients from the PUMCH CF cohort

Variable	All (*N* = 67)	Age at Diagnosis ≥ 18 y (*N* = 31)	Age at Diagnosis < 18 y (*N* = 36)	*P* value
**Age at diagnosis**,** years**	17.5 ± 8.8	24.5 ± 6.6	11.5 ± 5.3	< 0.001^a^
**Male**	35 (52.2)	15 (48.4)	20 (55.5)	0.558^b^
**Symptoms at diagnosis**				
Pulmonary	45 (70.3)	24 (80.0)	21 (63.6)	0.151^b^
Gastrointestinal	1 (1.6)	0	1 (3.0)	1.000^c^
Pulmonary and GI	17 (26.6)	6 (20.0)	11 (33.3)	0.234^b^
Male infertility	13 (92.9)	9 (90.0)	4 (100.0)	1.000^c^
**Presence of G970D**	24* (36.9)	14 (46.7)	10 (28.6)	0.132^b^
**PI (ever)**	22 (43.1)	6 (24.0)	16 (61.5)	0.007^b^
**Follow-up**,** years**	5.2 (2.7–8.1)	5.2 (2.7–7.6)	5.2 (2.7-8.0)	0.271^d^
**Lung transplantations**	2 (3.0)	0	2 (5.6)	0.495^c^
**Deaths**	3 (4.5)	0	3 (8.3)	0.243^c^
**Age at death or transplantation**,** years**	18.7 (17.2–19.3)	/	/	…
**Baseline characteristics**				
BMI, kg/m^2^	18.0 ± 3.5	19.8 ± 2.8	16.5 ± 3.4	< 0.001^a^
FEV_1_% predicted	67.1 (44.8–80.0)	66.4 (44.5–80.2)	67.1 (45.1–80.0)	0.329^d^
PEx	39 (60.9)	20 (64.5)	19 (57.6)	0.570^b^
PEx requiring hospitalization	20 (31.3)	9 (29.0)	11 (33.3)	0.711^b^
PA-positive results	44 (73.3)	21 (72.4)	23 (74.2)	0.876^b^
MRSA-positive results	6 (10.0)	3 (10.3)	3 (9.7)	1.000^c^
MABC-positive results	4 (6.7)	1 (3.4)	3 (9.7)	0.613^c^
* Burkholderia cenocepacia*-positive results	1 (1.7)	1(3.4)	0	0.483^c^
With ABPA	16 (23.9)	6 (24.0)	10 (27.7)	0.420^b^
CF-ABLE score	2.5 (2.0-5.4)	3.0 (2.0-5.5)	2.5 (2.0-5.1)	0.499^d^
3-year prognostic score	1.5 (0.5–2.9)	1.5 (0.5-3.0)	1.5 (0.5–2.6)	0.252^d^

The data are presented as No. (%), mean ± SD, or median (interquartile range). ABPA = allergic bronchopulmonary aspergillosis; BMI = body mass index; MRSA = methicillin-resistant *Staphylococcus aureus*; MABC = *Mycobacterium abscessus complex*; PA = *Pseudomonas aeruginosa*; PI = pancreatic exocrine insufficiency; PEx = pulmonary exacerbation; FEV_1_% = percent predicted forced expiratory volume in one second

* Three patients had homozygous variations in *CFTR* G970D

^a^ Student’s t test

^b^ Chi-square test

^c^ Fisher’s exact test

^d^ Wilcoxon rank-sum test

### Overall trajectory of lung function changes

**Fig. 3:**
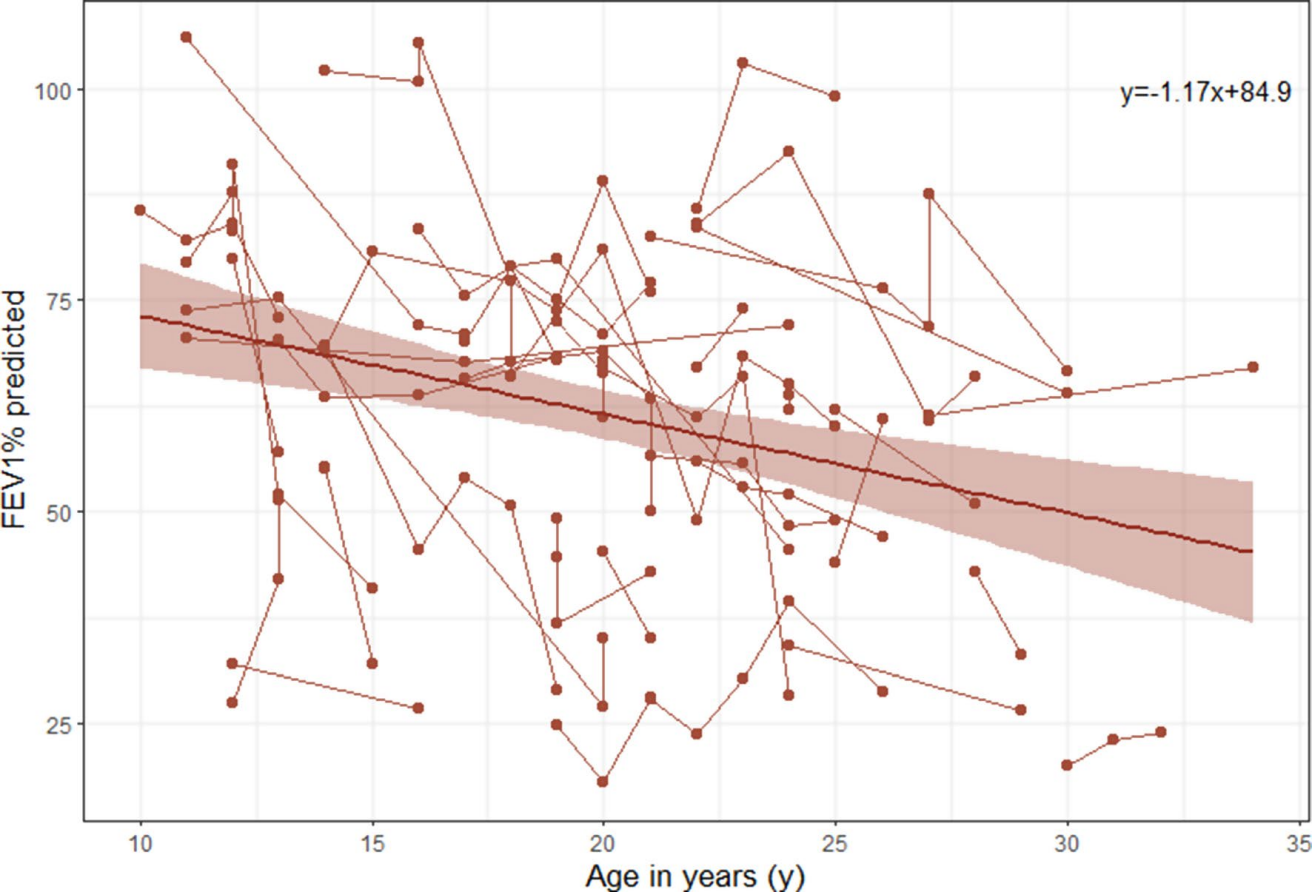
Graph showing the overall predicted trajectory of FEV_1_% predicted from mixed-effects model (red line), by age, and actual observed individual trajectories of each cystic fibrosis patient who was diagnosis ≤ 30 years in the study (red lines with dots)

For 45 patients who underwent at least two lung function tests taken from non-PEx period and separated by ≥ 3 months, their data was used for the progression analysis of CF (e-Table 41). The lung function showed significantly different trajectories at different ages. The decline rate of FEV_1_% predicted was relatively fast from 10 to 20 years of age but was gradually slow from 20 to 30 years of age. However, the trajectory showed an upward trend after the age of 30 (e-Fig. 1). Only 5 patients were over 30 years old at diagnosis, and three of those patients had the baseline FEV_1_% predicted greater than 80%. Therefore, sensitivity analysis was conducted to excluded the influence of these patients on the model. For patients ≤ 30 years of age at diagnosis, FEV_1_% predicted decreased 1.17% annually (Fig. 3).

### Clinical characteristics associated with change in lung function

The average annual change rate of FEV_1_% predicted was used as a dependent variable to construct a generalized linear model. In multivariable analysis, patients with better baseline FEV_1_% predicted have a greater annual rate of decline in FEV_1_% predicted (*P* = 0.02). Compared to patients without PEx (within 2 years after the date of diagnosis of CF), the FEV_1_% predicted changes in patients with PEx decreased at an average of -2.67% annually (Table 2). In the univariate analysis, as the diagnosis age increased, the annual rate of decline in FEV_1_% predicted decreased (*P* = 0.03). However, there was no statistical significance in multivariable analysis. Sex, BMI, PA infection, and other clinical characteristics were not significantly associated with changes in FEV_1_% predicted (e-Table 5).

**Table 2:** Generalized Linear Regression Model of the Annual rate of decline in FEV1% predicted

Characteristics	Univariate analysis		Multivariable analysis	
	Estimate (95% CI)	*P* value	Estimate (95% CI)	*P* value
Sex (reference, male)	-0.74 (-2.88 to 1.41)	0.51	-0.83 (-2.82 to 1.16)	0.40
Age at diagnosis	0.23 (0.03 to 0.42)	0.03	0.12 (-0.07 to 0.32)	0.21
Baseline FEV_1_% predicted	-0.06 (-0.11 to -0.01)	0.02	-0.06 (-0.11 to -0.01)	0.02
PEx (Yes/No)	-2.85 (-4.96 to -0.74)	0.01	-2.67 (-4.72 to -0.63)	0.01

PEx = pulmonary exacerbations; CI = confidence interval; FEV_1_% = percent predicted forced expiratory volume in one second

### Clinical characteristics associated with survival

**Fig. 4:**
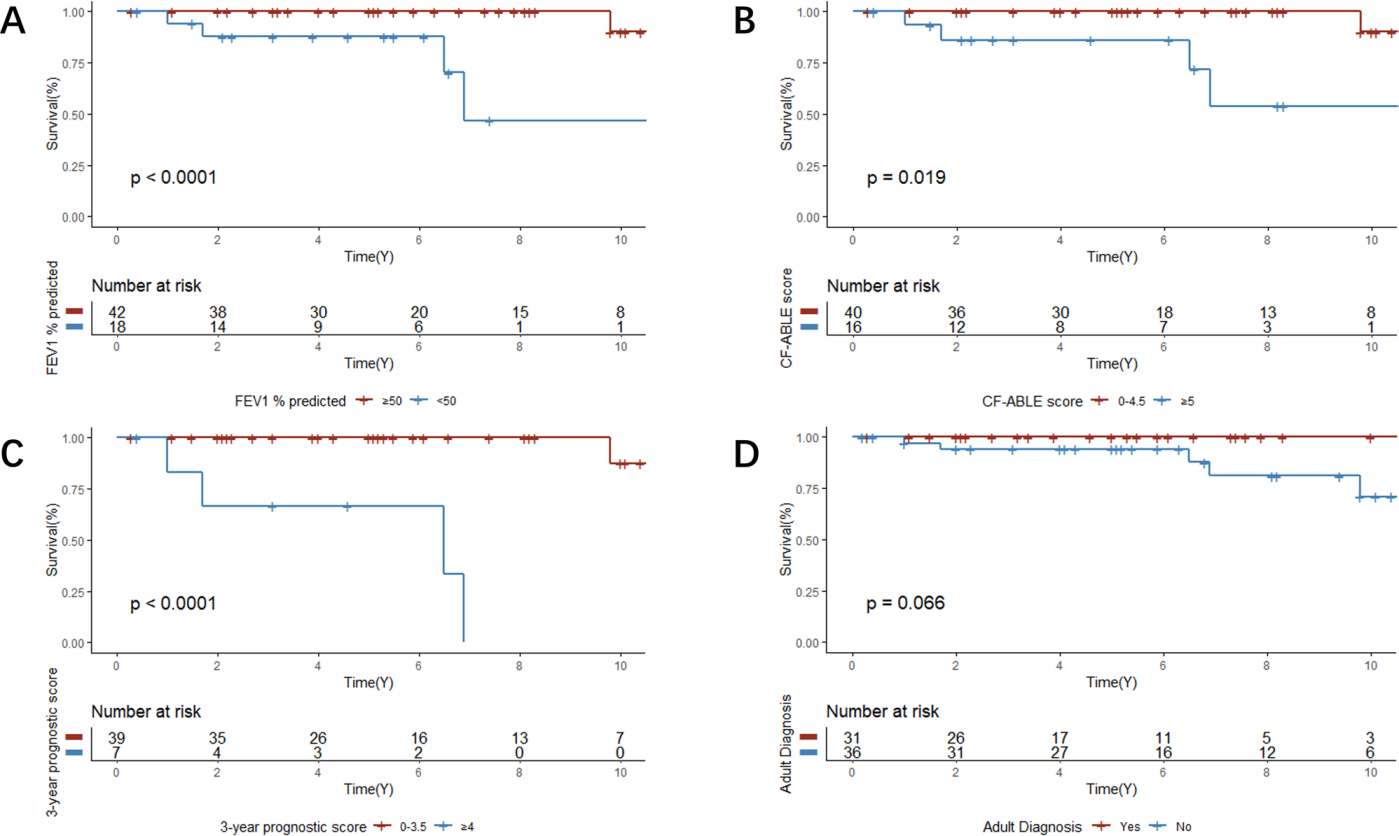
A-D, Kaplan–Meier survival curves for patients with cystic fibrosis. **A**, Survival curves by baseline FEV_1_% predicted. **B**, Survival curves by the CF-ABLE score. **C**, Survival curves by the 3-year prognostic score. **D**, Survival curves by whether the patient was adult-diagnosed

The cumulative survival rates at 5 years and 10 years after the diagnosis were 96.7% and 80.6%, respectively. The 5-year cumulative survival rates of patients with baseline FEV_1_% predicted ≥ 50% and baseline FEV_1_% predicted < 50% were 100.0% and 87.8%, respectively (*P* < 0.0001) (Fig. 4A). The 5-year cumulative survival rates of patients with CF-ABLE scores from 0 to 4.5 (low to moderate score) and CF-ABLE score ≥ 5 (high score) were 100.0% and 86.2%, respectively (*P* = 0.019) (Fig. 4B). The 5-year cumulative survival rates of patients with 3-year prognostic score from 0 to 3.5 (low to moderate score) and 3-year prognostic score ≥ 4 (high score) were 100.0% and 66.7%, respectively (*P* < 0.0001) (Fig. 4C).

The 5-year cumulative survival rates of patients who were diagnosed with CF during adulthood and childhood were 100.0% and 94.0%, respectively (*P* = 0.066) (Fig. 4D). The 5-year cumulative survival rates of patients having CF but without ABPA and those having CF with ABPA were 97.7% and 93.8%, respectively (*P* = 0.053) (e-Fig. 2). Other factors such as BMI, PExs requiring hospitalization, PA infection, and the presence of the G970D mutation did not show significant differences in the log-rank test.

## Discussion

This study used the PUMCH CF cohort to investigate determinants of progression and mortality in Chinese patients with CF. Overall, baseline FEV_1_% predicted and PEx were associated with progression in Chinese patients with CF, while baseline FEV_1_% predicted, CF-ABLE score, and 3-year prognostic score were associated with mortality.

CF was previously considered to be a disease affecting Caucasians. However, there is increasing evidence that CF is present in Asia, Africa, the Middle East and Latin America, albeit at a lower incidence than in White persons [1, 17]. Studies from multiple centers suggest that the clinical and genetic characteristics of Chinese patients with CF are different from those of the Caucasians [5, 6, 18–21]. In this study, Chinese patients with CF mainly displayed pulmonary symptoms (96.9%) with fewer gastrointestinal symptoms such as PI (43.1%). However, in the Caucasians, PI affects approximately 80% of those with CF [1]. Furthermore, G970D is the most frequent *CFTR* mutation among Chinese patients, with an allele frequency of 20.8%. F508del, the most common pathogenic mutation in Caucasians with CF comprising approximately 85% of all CF-related alleles in the United States, is rare in Chinese patients [1, 18].

Compared to patients diagnosed with CF in childhood, adult-diagnosed patients exhibit a milder phenotype. In our study, adult-diagnosed patients had a lower proportion of PI (24.0% vs. 61.5%) and a higher BMI (19.8 vs. 16.5 kg/m^2^), which is consistent with previous findings in Caucasians [14]. One possible explanation is that patients who are younger at diagnosis may have more severe mutations, which may lead to more severe clinical phenotypes and earlier clinic visits. However, the proportion of G970D mutations in the two groups (adult-diagnosed and childhood-diagnosed) did not support this explanation. G970D, as a Class III variant of *CFTR*, which would seriously affect the function of CFTR, was actually more common in adult-diagnosed patients (46.7% vs. 28.6%) [22]. As most of the *CFTR* variants were only observed in patients of Chinese origin, the variant classification is not clear, and it is therefore difficult to determine the relationship between *CFTR* mutation type and phenotype severity.

Our study used a mixed-effects model to describe the trajectory of FEV_1_% predicted changes with age in Chinese patients with CF. In the initial mixed-effects model (including all 45 patients who have at least two lung function tests taken from non-PEx period and separated by ≥ 3 months), the trajectory of FEV_1_% predicted shows an upward trend after the 30 years of age. This is because there are too few patients diagnosed over the age of 30 (only 5 patients), and some of these patients have good and stable lung function. Subsequently, we excluded these 5 patients for sensitivity analysis, and found that the decline in FEV_1_% predicted of the remaining patients could be fitted with a straight line as age changed. Regardless of whether the patient is an adult at the time of diagnosis, the rate of decline in FEV_1_% predicted is -1.17%/y. This rate of decline was slightly slower than that observed in Caucasians. Keating et al. reported that the rate of annual change in FEV_1_% predicted were -2.04% from 12 to 17 years of age and -1.13% from 18 to 65 years of age in American patients with CF [13]. And the data from adult patients in the United Kingdom registry showed an overall annual decline in FEV_1_% predicted of -1.52%/y [23]. Perhaps due to the small number of patients and the failure to include patients diagnosed over 30 years old, our study did not find a difference in the rate of FEV_1_% predicted decline between adult and childhood diagnosed patients.

Higher baseline FEV_1_% predicted, and occurrence of PEx have been found to be associated with progression in Chinese patients with CF. Previous studies have shown that the higher the FEV_1_% predicted at baseline, the faster the decline in lung function [13]. This is because the decline in FEV_1_% predicted tends to be less steep once lung function declines into the moderate to severely reduced ranges. PEx can reduce lung function and increase mortality, seriously affecting the prognosis of patients with CF. Kerem et al. [9] found that PI was associated with a decline in FEV_1_% predicted in patients with CF. In this study, no relationship was found between PI and decline in lung function. This may be related to the use of fecal Sudan III, which has a lower sensitivity, for determining PI in this study. It is worth noting that with the extension of follow-up time, the incidence of PI significantly increased compared to our study conducted 5 years ago (43.1% vs. 14.1%) [6]. In the future, with the extension of follow-up time and the popularization of fecal elastase test, more PI patients will be detected.

The survival rate of patients with CF in China differs from that of the Caucasians, with a higher survival rate in adult-diagnosed patients and a lower survival rate in childhood-diagnosed patients. In our study, the 10-year survival rates of patients with CF who were diagnosed in adulthood or childhood were 100.0% and 70.9%, respectively. According to Keating et al. [13], the 10-year survival rate of adult patients with CF in the United States is 76%. However, it has been reported that the survival rate of pediatric patients is higher than that of adults in the United States, and the death of children with CF in the United Kingdom is very rare; therefore, the survival rate of Caucasian children with CF should be higher [24, 25]. CFTR modulator therapy significantly reduced the mortality rate of patients with CF [1]. In contrast to Caucasian populations, which are generally treated with CFTR modulators (82%, according to Annual Data Report 2022 of Cystic Fibrosis Foundation Patient Registry), the current treatments for Chinese patients mainly include airway clearance, long-term oral macrolides, pancreatic enzyme replacement, and antibiotic therapy during PEx [2]. In December 2020, the U.S. Food and Drug Administration approved Kalydeco (ivacaftor) for patients with CF carrying at least one copy of the G970D mutation. In the future, with the use of CFTR modulators in Chinese patients, the survival rate of Chinese patients with CF is expected to improve.

Baseline FEV_1_% predicted < 50%, and high CF-ABLE and 3-year prognostic scores were associated with mortality in Chinese patients with CF. McCarthy et al. [11] identified that 52% of FEV_1_% predicted was an accurate predictor of outcome and identified high-risk patients earlier. In this study, we selected a close value of 50% as the cutoff point and also found a relationship between FEV_1_% predicted and mortality. The CF-ABLE score and 3-year prognostic score for are tools to predict the risk of death or lung transplantation in patients with CF in the Caucasian population [11, 12]. Our study found that these two prediction tools are equally applicable to Chinese patients with CF.

This study had several limitations. First, most patients included in this study were ≤ 30 years old, and lung function data for those > 30 years were lacking; therefore, the changes in lung function for patients > 30 years cannot be well described. Second, owing to the relatively small number of patients with CF in China, the absolute number of deaths or lung transplant recipients is also relatively small, the conclusions may be influenced by accidental factors. Therefore, the analysis results in this section are exploratory. Notably, lung function is also an indicator of the CF-ABLE and 3-year prognostic scores. Therefore, it was difficult to evaluate the impact of confounding factors on patient mortality. Third, as this was a single-center study, most of the included patients were from northern and eastern China. Shen et al. [18] found that the G970D variant was more common in northern and eastern China. The differences in regional distribution of the variation may have led to an overestimation of the proportion of G970D variation in this study. Finally, there are likely many undiagnosed patients in China given the incidence and the size of the Chinese population. The people that actually come to medical attention and receive a diagnosis of CF in China are a very unique population, which requires attention when generalizing our data to the Chinese population. However, several previous studies have summarized the clinical and genetic characteristics of all published Chinese patients with CF, and their results are consistent with this study [6, 19]. Therefore, we believe that the patients in this study can represent all patients with CF in China to a certain extent.

## Conclusion

Chinese patients with CF were a rare and relatively unknown population in the past. This is the first study to analyze the progression and mortality of Chinese patients with CF and their determinant factors. Baseline FEV_1_% predicted is associated with progression and mortality. PEx accelerates the rate of decline in lung function. The CF-ABLE and 3-year prognostic scores can be used to predict poor prognosis in Chinese patients with CF.

## Electronic supplementary material

Below is the link to the electronic supplementary material.

Supplementary Material 1

## Data Availability

The raw data that support the findings of this study are not publicly available due individual privacy but are available from the corresponding author upon reasonable request.
