# Supplementary material for: Progression and mortality of patients with cystic fibrosis in China
Source: Orphanet J Rare Dis. 2025 Jan 7;20:6. doi: 10.1186/s13023-024-03522-1 (PMC11705856; doi:10.1186/s13023-024-03522-1)
Supplement: Supplementary file 1 — Supplementary Material 1 [file 13023_2024_3522_MOESM1_ESM.docx]

**e-Table 1 *CFTR* mutations and sweat chloride values of patients in PUMCH CF cohort**

| **Patient No.** | **Sweat chloride value, mmol/L^#^** | **Nucleotide change** | **Amino acid change** | **Genotype** | **Pathogenic significance*** | **ACMG Guideline** | | **Reference** |
| --- | --- | --- | --- | --- | --- | --- | --- | --- |
|  |  |  |  |  |  | **Class** | **Evidence** |  |
| 1 | 132 | c.2052dupA | p.Gln685ThrfsX4 | Compound heterozygous | CF-causing | Path | PVS1+PS4+PM2 | ^1^ |
|  |  | △E18-E20  (c.2909-?_3367 + ?del) | p.Gly980_Thr1112delinsGly |  | NA | Path | PVS1+PM2+PP3 |  |
| 2 | 95 | c.3700A>G | p.Ile1234Val | Compound heterozygous | CF-causing | Path | PS3+PS4 | ^2^ |
|  |  | c.959-960insA | p.Ser321IlefsX42 |  | NA | Path | PVS1+PM2+PM3 |  |
| 3 | 218 | c.2909G>A | p.Gly970Asp | Compound heterozygous | CF-causing | Path | PS3+PS4+PM2+PM3+PP3 | ^3^ |
|  |  | c.3883_3886delATTT | p.Ile1295PhefsX32 |  | CF-causing | Path | PVS1+PM2+PM3 |  |
| 4 | 138 | c.2909G>A | p.Gly970Asp | Compound heterozygous | CF-causing | Path | PS3+PS4+PM2+PM3+PP3 | ^2^ |
|  |  | c.263T>G | p.Leu88X |  | CF-causing | Path | PVS1+ PM2+PM3 |  |
| 5 | 68 | c.374T>C | p.Ile125Thr | Heterozygous | NA | VUS | PP2+PP3+BS1 | Current |
| 6 | 80 | c.293A>G | p.Gln98Arg | Compound heterozygous | CF-causing | Path | PS3+PS4 | ^3^ |
|  |  | c.2353C>T | p.Arg785X |  | CF-causing | Path | PVS1+ PM2+PM3 |  |
| 7 | 143 | c.2909G>A | p.Gly970Asp | Compound heterozygous | CF-causing | Path | PS3+PS4+PM2+PM3+PP3 | ^3^ |
|  |  | c.3068T>G | p.Ile1023Arg |  | NA | Path | PS2+PM1+PM2+PM3+PP2 |  |
| 8 | 154 | c.293A>G | p.Gln98Arg | Homozygous | CF-causing | Path | PS3+PS4 | ^3^ |
| 9 | 164 | c.2909G>A | p.Gly970Asp | Compound heterozygous | CF-causing | Path | PS3+PS4+PM2+PM3+PP3 | ^3^ |
|  |  | c.2997_3000delAATT | p.Ile1000X |  | CF-causing | Path | PVS1+PS4+PM2 |  |
| 10 | 128 | c.2909G>A | p.Gly970Asp | Compound heterozygous | CF-causing | Path | PS3+PS4+PM2+PM3+PP3 | ^1^ |
|  |  | △E7-E11  (c.744-?_1584+?del) | p.Arg248_Glu528delinsArgfsX11 |  | NA | Path | PVS1+PM2+PP3 |  |
| 11 | 66 | c.95T>C | p.Leu32Pro | Compound heterozygous | NA | VUS | PM2+PP2+PP3 | ^1^ |
|  |  | c.1657C>T | p.Arg553X |  | CF-causing | Path | PVS1+PM2+PM3 |  |
| 12 | 118 | c.2909G>A | p.Gly970Asp | Homozygous | CF-causing | Path | PS3+PS4+PM2+PM3+PP3 | ^3^ |
| 13 | 109 | c.2909G>A | p.Gly970Asp | Compound heterozygous | CF-causing | Path | PS3+PS4+PM2+PM3+PP3 | ^2^ |
|  |  | c.1997T>G | p.Leu666X |  | NA | Path | PVS1+PM2+PP5 |  |
| 14 | 114 | c.293A>G | p.Gln98Arg | Compound heterozygous | CF-causing | Path | PS3+PS4 | ^1^ |
|  |  | c.558C>G | p.Asn186Lys |  | NA | VUS | PM1+PM2+PP2 |  |
| 15 | 142 | △E20  (c.3140-454_c.3367+249del931ins13) | p.Arg1048_Gly1123del | Homozygous | NA | Path | PVS1+PM2+PP3 | ^4^ |
| 16 | 34/41 | c.214G>A | p.Ala72Thr | Compound heterozygous | NA | LP | PM1+PM2+PM3+PP2+PP3 | ^5^ |
|  |  | c.3406G>A | p.Ala1136Thr |  | NA | LP | PM2+PM3+PP2+PP3+PP5 |  |
| 17 | 186 | c.2909G>A | p.Gly970Asp | Homozygous | CF-causing | Path | PS3+PS4+PM2+PM3+PP3 | ^3^ |
| 18 | 199 | c.1766+5G>T | - | Homozygous | CF-causing | Path | PS3+PS4+PM2+PM3+PP3 | ^3^ |
| 19 | 108 | c.2909G>A | p.Gly970Asp | Compound heterozygous | CF-causing | Path | PS3+PS4+PM2+PM3+PP3 | ^3^ |
|  |  | c.2125C>T | p.Arg709X |  | CF-causing | Path | PVS1+PM2+PP3 |  |
| 20 | 105 | c.3635delT | p.Val1212AlafsX16 | Heterozygous | NA | Path | PVS1+PM2+PP5 | ^2^ |
| 21 | 69 | c.1666A>G | p.Ile556Val | Heterozygous | NA | Benign | BA1+BS2 | ^1^ |
| 22 | 151 | c.3635delT | p.Val1212AlafsX16 | Compound heterozygous | NA | Path | PVS1+PM2+PP5 | ^3^ |
|  |  | c.607A>T | p.Ile203Phe |  | NA | LP | PM1+PM2+PP2+PP3 |  |
| 23 | ND | c.2909G>A | p.Gly970Asp | Compound heterozygous | CF-causing | Path | PS3+PS4+PM2+PM3+PP3 | ^3^ |
|  |  | c.1657C>T | p.Arg553X |  | CF-causing | Path | PVS1+PM2+PM3 |  |
| 24 | 154 | c.1679+2T>C | - | Compound heterozygous | CF-causing | Path | PVS1+PM2+PP5 | ^1,3^ |
|  |  | c.2658-1G>C | - |  | CF-causing | Path | PVS1+PM2+PP5 |  |
| 25 | 124 | △E2-E3  (c.54-?_273+?del) | - | Heterozygous | NA | Path | PVS1+PM2+PP3 | ^3^ |
| 26 | 192 | c.2909G>A | p.Gly970Asp | Compound heterozygous | CF-causing | Path | PS3+PS4+PM2+PM3+PP3 | ^3^ |
|  |  | c.2125C>T | p.Arg709X |  | CF-causing | Path | PVS1+PM2+PP3 |  |
| 27 | 210 | c.405_406dupAC | p.Leu136HisfsX18 | Compound heterozygous | NA | Path | PVS1+PM2+PM3 | ^3^ |
|  |  | c.1388G>A | p.Gly463Asp |  | NA | LP | PM1+PM2+PM3+PP2+PP3 |  |
| 28 | 131 | c.2909G>A | p.Gly970Asp | Compound heterozygous | CF-causing | Path | PS3+PS4+PM2+PM3+PP3 | ^6^ |
|  |  | c.1210-3C>G | - |  | NA | Path | PVS1+PM2+PM3 |  |
| 29 | 133 | c.2909G>A | p.Gly970Asp | Compound heterozygous | CF-causing | Path | PS3+PS4+PM2+PM3+PP3 | ^3^ |
|  |  | c.2547C>A | p.Tyr849X |  | CF-causing | Path | PVS1+PM2+PM3 |  |
| 30 | 86 | c.3472C>T | p.Arg1158X | Compound heterozygous | CF-causing | Path | PVS1+PM2+PM3 | Current |
|  |  | c.1209+1G>C | - |  | CF-causing | Path | PVS1+PM2+PM3 |  |
| 31 | 111 | c.1766+5G>T | - | Homozygous | CF-causing | Path | PS3+PS4+PM2+PM3+PP3 | ^7^ |
| 32 | 81 | ND | - | - | - | - | - | - |
| 33 | 63 | c.3196C>A | p.Arg1066Ser | Heterozygous | NA | LP | PM1+PM2+PM5+PP2+PP3 | Current |
| 34 | 81 | c.293A>G | p.Gln98Arg | Homozygous | CF-causing | Path | PS3+PS4 | ^1^ |
| 35 | 99 | c.2909G>A | p.Gly970Asp | Compound heterozygous | CF-causing | Path | PS3+PS4+PM2+PM3+PP3 | ^3^ |
|  |  | c.595C>T | p.His199Tyr |  | CF-causing | Path | PS3+PM2+PM3+PP2+PP3 |  |
| 36 | 120 | c.3700A>G | p.Ile1234Val | Compound heterozygous | CF-causing | Path | PS3+PS4 | ^2^ |
|  |  | c.959_960insA | p.Ser321IlefsX42 |  | NA | Path | PVS1+PM2+PM3 |  |
| 37 | ND | c.263T>G | p.Leu88X | Compound heterozygous | CF-causing | Path | PVS1+ PM2+PM3 | ^8^ |
|  |  | c.1766+5G>T | - |  | CF-causing | Path | PS3+PS4+PM2+PM3+PP3 |  |
| 38 | 111 | c.2909G>A | p.Gly970Asp | Compound heterozygous | CF-causing | Path | PS3+PS4+PM2+PM3+PP3 | ^2^ |
|  |  | c.263T>G | p.Leu88X |  | CF-causing | Path | PVS1+ PM2+PM3 |  |
| 39 | 112 | c.2909G>A | p.Gly970Asp | Compound heterozygous | CF-causing | Path | PS3+PS4+PM2+PM3+PP3 | ^2^ |
|  |  | c.263T>G | p.Leu88X |  | CF-causing | Path | PVS1+ PM2+PM3 |  |
| 40 | 36/38 | c.3718-2477C>T | - | Homozygous | CF-causing | Path | PS3+PM2+PM3+PP1+PP5 | Current |
| 41 | ND | c.2909G>A | p.Gly970Asp | Compound heterozygous | CF-causing | Path | PS3+PS4+PM2+PM3+PP3 | ^3^ |
|  |  | c.1716C>A | p.Asp572Glu |  | NA | LP | PM1+PM2+PM5+PP2+PP3 |  |
| 42 | 136 | c.2909G>A | p.Gly970Asp | Compound heterozygous | CF-causing | Path | PS3+PS4+PM2+PM3+PP3 | Current |
|  |  | c.3909C>G | p.Asn1303Lys |  | CF-causing | Path | PS3+PS4 |  |
| 43 | 88 | c1925C>G | p.Ser642X | Compound heterozygous | NA | Path | PVS1+PM2+PM3+PP3 | Current |
|  |  | c.3G>A | p.Met1Ile |  | NA | Path | PVS1+PS1+PS4+PM2 |  |
| 44 | ND | c.262_266delTTATA | p.Leu88PhefsX21 | Compound heterozygous | NA | Path | PVS1+ PM2+PM3 | Current |
|  |  | c.2547C>A | p.Tyr849X |  | CF-causing | Path | PVS1+PM2+PM3 |  |
| 45 | ND | c.223C>T | p.Arg75X | Compound heterozygous | CF-causing | Path | PVS1+PM2+PM3 | Current |
|  |  | c.3763T>C | p.Ser1255Pro |  | CF-causing | Path | PS3+PM1+PM2+PM3+PP3 |  |
| 46 | 156 | c.2909G>A | p.Gly970Asp | Compound heterozygous | CF-causing | Path | PS3+PS4+PM2+PM3+PP3 | ^2^ |
|  |  | c.1521_1523delCTT | p.Phe508del |  | CF-causing | Path | PS3+PS4 |  |
| 47 | 109 | c.2909G>A | p.Gly970Asp | Compound heterozygous | CF-causing | Path | PS3+PS4+PM2+PM3+PP3 | Current |
|  |  | c.1344_1347delAGAA | p.Ile448IlefsX20 |  | NA | Path | PVS1+PM2+PP3 |  |
| 48 | 108 | c.1521_1523delCTT | p.Phe508del | Compound heterozygous | CF-causing | Path | PS3+PS4 | Current |
|  |  | c.1766+5G>T | - |  | CF-causing | Path | PS3+PS4+PM2+PM3+PP3 |  |
| 49 | ND | c.2977G>T | p.Asp993Tyr | Compound heterozygous | NA | LP | PM2+PM3+PP2+PP3+PP5 | Current |
|  |  | c.3140-26A>G | - |  | CF-causing | Path | PS3+PS4+PM2+PM3+PP3 |  |
|  |  | c.1210-11T>G | - |  | Varying clinical consequences | Path | PS3+PM2+PM3+PP3+PP5 |  |
| 50 | 110 | ND | - | - | - | - | - | - |
| 51 | 36/39 | c.647G>A | p.Trp216X | Compound heterozygous | CF-causing | Path | PVS1+PM2+PM3 | Current |
|  |  | c.1210-11T>G | - |  | Varying clinical consequences | Path | PS3+PM2+PM3+PP3+PP5 |  |
| 52 | 142 | c.2036G>A | p.Trp679X | Compound heterozygous | CF-causing | Path | PVS1+PS4+PM2 | Current |
|  |  | c.567C>A | p.Asn189Lys |  | NA | LP | PM1+PM2+PM3+PP2+PP3 |  |
| 53 | 112 | c.2909G>A | p.Gly970Asp | Compound heterozygous | CF-causing | Path | PS3+PS4+PM2+PM3+PP3 | ^2^ |
|  |  | c.1521_1523delCTT | p.Phe508del |  | CF-causing | Path | PS3+PS4 |  |
| 54 | 84 | c.2909G>A | p.Gly970Asp | Compound heterozygous | CF-causing | Path | PS3+PS4+PM2+PM3+PP3 | ^2^ |
|  |  | c.2374C>T | p.Arg792X |  | CF-causing | Path | PVS1+PM2+PM3 |  |
| 55 | 108 | c.264-268delATATT | p.Leu88PhefsX21 | Compound heterozygous | NA | Path | PVS1+PM2+PM3 | Current |
|  |  | c.400A>G | p.Arg134Gly |  | NA | LP | PM2+PM3+PP2+PP3 |  |
| 56 | 129 | c.2909G>A | p.Gly970Asp | Homozygous | CF-causing | Path | PS3+PS4+PM2+PM3+PP3 | ^3^ |
| 57 | ND | c.264-268delATATT | p.Leu88PhefsX21 | Compound heterozygous | NA | Path | PVS1+PM2+PM3 | Current |
|  |  | c.3717G>A | p.Arg1239Arg |  | CF-causing | Path | PS3+PS4+PM2+PP3 |  |
| 58 | 136 | c.2909G>A | p.Gly970Asp | Compound heterozygous | CF-causing | Path | PS3+PS4+PM2+PM3+PP3 | Current |
|  |  | c.3745G>C | p.Gly1249Arg |  | NA | Path | PS1+PS4+PM1+PM2+PM5 |  |
| 59 | 124 | c.2374C>T | p.Arg792X | Compound heterozygous | CF-causing | Path | PVS1+PM2+PM3 | ^7^ |
|  |  | △E4-E11  (c.274-?_1584+?del) | - |  | NA | Path | PVS1+PM2+PP3 |  |
| 60 | 118 | c.2036G>A | p.Trp679X | Compound heterozygous | CF-causing | Path | PVS1+PS4+PM2 | ^7^ |
|  |  | c.164+2T>C | - |  | CF-causing | Path | PVS1+PS4+PM2 |  |
| 61 | 112 | c.233dupT | p.Trp79LeufsX32 | Compound heterozygous | CF-causing | Path | PVS1+PM2+PM3 | ^7^ |
|  |  | △E20  (c.3140-?_3367+?del) | p.Arg1048_Gly1123del |  | NA | Path | PVS1+PM2+PP3 |  |
| 62 | 126 | c.1000C>T | p.Arg334Trp | Homozygous | CF-causing | Path | PS3+PM1+PM2+PM3+PP3 | Current |
| 63 | 20/32 | c.3718-2477C>T | - | Homozygous | CF-causing | Path | PS3+PM2+PM3+PP1+PP5 | Current |
| 64 | 108 | c.595C>T | p.His199Tyr | Compound heterozygous | CF-causing | Path | PS3+PM2+PM3+PP2+PP3 | Current |
|  |  | c.2060_2061del | p.Phe687X |  | NA | Path | PVS1+PM2+PM3 |  |
| 65 | 147 | c.595C>T | p.His199Tyr | Compound heterozygous | CF-causing | Path | PS3+PM2+PM3+PP2+PP3 | Current |
|  |  | c.2374C>T | p.Arg792X |  | CF-causing | Path | PVS1+PM2+PM3 |  |
| 66 | 28/33 | c.2997_3000del | p.Ile1000X | Compound heterozygous | CF-causing | Path | PVS1+PS4+PM2 | Current |
|  |  | c.2936A>C | p.Asp979Ala |  | NA | Path | PS3+PS4+PM1+PM2+PM5 |  |
| 67 | 152 | c.223C>T | p.Arg75X | Compound heterozygous | CF-causing | Path | PVS1+PM2+PM3 | Current |
|  |  | c.865A>T | p.Arg289X |  | CF-causing | Path | PVS1+PM2+PP5 |  |

ACMG: American College of Medical Genetics; LP: likely pathogenic; NA: not available in CFTR2; ND: not done; Path: pathogenic; VUS: variant of uncertain significance.

^#^ Each patient undergoes two sweat chloride tests. If the results are both greater than 60mmol/L, record the low value. Otherwise, record the results of two separate tests.

* According to The Clinical and Functional TRanslation of CFTR (CFTR2); available at <http://cftr2.org.>

**References**

1. Liu Y, Wang L, Tian X, et al. Characterization of gene mutations and phenotypes of cystic fibrosis in Chinese patients. *Respirology.* 2015;20(2):312-318.

2. Tian X, Liu Y, Yang J, et al. p.G970D is the most frequent CFTR mutation in Chinese patients with cystic fibrosis. *Hum Genome Var.* 2016;3:15063.

3. Liu K, Xu W, Xiao M, et al. Characterization of clinical and genetic spectrum of Chinese patients with cystic fibrosis. *Orphanet J Rare Dis.* 2020;15(1):150.

4. Liu K, Liu Y, Li X, Xu KF, Tian X, Zhang X. A novel homozygous complex deletion in CFTR caused cystic fibrosis in a Chinese patient. *Mol Genet Genomics.* 2017;292(5):1083-1089.

5. Guo J, He R, Mao ZQ. Case Report: White Colored Stool: An Early Sign of Cystic Fibrosis in Infants. *Front Pediatr.* 2021;9:656584.

6. Zhao X, Liu K, Xu W, et al. Novel mutation c.1210-3C > G in cis with a poly-T tract of 5T affects CFTR mRNA splicing in a Chinese patient with cystic fibrosis. *Front Med.* 2022;16(1):150-155.

7. Shen Y, Tang X, Chen Q, et al. Genetic spectrum of Chinese children with cystic fibrosis: comprehensive data analysis from the main referral centre in China. *J Med Genet.* 2022;60(3):310-315.

8. Shen Y, Liu J, Zhong L, et al. Clinical Phenotypes and Genotypic Spectrum of Cystic Fibrosis in Chinese Children. *J Pediatr.* 2016;171:269-276.e261.

**e-Table 2** **Parameters of model fits statistics (Smaller is better)**

Models shown in the table were constructed to evaluate the trajectory of FEV_1_% predicted over time: 1) and 2) Linear model with random intercepts (age at pulmonary function test as continuous linear variable); 3) and 4) Linear model with random intercepts and slopes; 5) and 6) Quadratic model (^2) with random intercepts and slopes (quadratic relationship between age and FEV1% predicted); 7) and 8) Cubic model (^3) with random intercepts and slopes (cubic relationship between age and FEV1% predicted); 9) and 10) Quadratic spline model with three knots at quantiles; 11) and 12) Cubic spline model with three knots at quantiles; 13) and 14) Quadratic spline model with one knot at median; 15) and 16) Cubic spline model with one knot at median.

| **Model type** | **AIC** | **BIC** | **-2Log-likelihood** |
| --- | --- | --- | --- |
| Linear model A* with random intercepts | 1240.3 | 1252.4 | -616.15 |
| Linear model B* with random intercepts | 1232.7 | 1247.8 | -611.33 |
| Linear model A with random intercepts and random slopes | 1229.0 | 1247.0 | -608.47 |
| Linear model B with random intercepts and random slopes | 1220.3 | 1241.5 | -603.18 |
| Quadratic model (^2) A with random intercepts and slopes | 1227.0 | 1248.2 | -606.52 |
| Quadratic model (^2) B with random intercepts and slopes | 1218.4 | 1242.5 | -601.20 |
| Cubic model (^3) A with random intercepts and slopes | 1228.7 | 1252.8 | -606.34 |
| Cubic model (^3) B with random intercepts and slopes | 1220.3 | 1247.5 | -601.16 |
| Quadratic spline model A with three knots at quantiles (25th, 50th, and 75th) | 1233.8 | 1267.0 | -605.91 |
| Quadratic spline model B with three knots at quantiles (25th, 50th, and 75th) | 1225.6 | 1261.8 | -600.80 |
| Cubic spline model A with two knots at quantiles (25th, 50th, and 75th) | 1230.1 | 1257.2 | -606.03 |
| Cubic spline model B with two knots at quantiles (25th, 50th, and 75th) | 1221.8 | 1252.0 | -600.92 |
| Quadratic spline model A with one knot at median quantile | 1230.5 | 1257.7 | -606.27 |
| Quadratic spline model B with one knot at median quantile | 1222.3 | 1252.5 | -601.14 |
| Cubic spline model A with one knot at median quantile | 1227.1 | 1248.2 | -606.55 |
| Cubic spline model B with one knot at median quantile | 1218.5 | 1242.6 | -601.23 |

model A*: individual variation was random effect.

model B*: age at diagnosis and individual variation were random effects.


**e-Table 3**. **Parameters of model fits statistics in sensitivity analysis (Smaller is better)**

Models shown in the table were constructed to evaluate the trajectory of FEV1% predicted over time in sensitivity analysis: 1) and 2) Linear model with random intercepts (age at pulmonary function test as continuous linear variable); 3) and 4) Linear model with random intercepts and slopes; 5) and 6) Quadratic model (^2) with random intercepts and slopes (quadratic relationship between age and FEV1% predicted); 7) and 8) Cubic model (^3) with random intercepts and slopes (cubic relationship between age and FEV1% predicted); 9) and 10) Quadratic spline model with three knots at quantiles; 11) and 12) Cubic spline model with three knots at quantiles; 13) and 14) Quadratic spline model with one knot at median; 15) and 16) Cubic spline model with one knot at median.

| Model type | AIC | BIC | -2Log-likelihood |
| --- | --- | --- | --- |
| Linear model A* with random intercepts | 1101.2 | 1112.9 | -546.63 |
| Linear model B* with random intercepts | 1093.4 | 1108.0 | -541.72 |
| Linear model A with random intercepts and random slopes | 1097.8 | 1115.3 | -542.93 |
| Linear model B with random intercepts and random slopes | 1089.0 | 1109.3 | -537.47 |
| Quadratic model (^2) A with random intercepts and slopes | 1098.9 | 1119.2 | -542.43 |
| Quadratic model (^2) B with random intercepts and slopes | 1090.2 | 1113.5 | -537.12 |
| Cubic model (^3) A with random intercepts and slopes | 1100.5 | 1123.8 | -542.27 |
| Cubic model (^3) B with random intercepts and slopes | 1092.2 | 1118.3 | -537.10 |
| Quadratic spline model A with three knots at quantiles (25th, 50th, and 75th) | 1103.5 | 1135.5 | -540.76 |
| Quadratic spline model B with three knots at quantiles (25th, 50th, and 75th) | 1095.9 | 1130.8 | -535.96 |
| Cubic spline model A with two knots at quantiles (25th, 50th, and 75th) | 1101.9 | 1128.0 | -541.93 |
| Cubic spline model B with two knots at quantiles (25th, 50th, and 75th) | 1093.8 | 1122.9 | -536.92 |
| Quadratic spline model A with one knot at median quantile | 1101.8 | 1128.0 | -541.92 |
| Quadratic spline model B with one knot at median quantile | 1093.5 | 1122.5 | -536.73 |
| Cubic spline model A with one knot at median quantile | 1099.0 | 1119.4 | -542.52 |
| Cubic spline model B with one knot at median quantile | 1090.4 | 1113.6 | -537.20 |

model A*: individual variation was random effect.

model B*: age at diagnosis and individual variation were random effects.


**e-Table 4 Baseline Characteristics of 45 Patients for Progression Analysis**

| **Variable** | **All (N=45)** | **Age at Diagnosis ≥ 18 y (N=25)** | **Age at Diagnosis < 18 y (N=20)** | ***P* value** |
| --- | --- | --- | --- | --- |
| Age at diagnosis, years | 19.6±7.8 | 24.7±6.6 | 13.1±2.8 | < 0.001^a^ |
| Male | 23（51.1） | 12（48.0） | 11（55.0） | 0.641^b^ |
| Symptoms at diagnosis |  |  |  | 0.469^b^ |
| Pulmonary | 31（70.5） | 18（75.0） | 13（65.0） | ... |
| Pulmonary and GI | 13（29.5） | 6（25.0） | 7（35.0） | ... |
| Presence of G970D |  |  |  | 0.107^b^ |
| Yes | 19*（43.2） | 13（54.2） | 6（30.0） | ... |
| No | 25（56.8） | 11（45.8） | 14（70.0） | ... |
| PI (ever) | 19（50.0） | 5（25.0） | 14（77.8） | 0.001^b^ |
| Follow-up, years | 5.9（3.2–8.3） | 5.0（2.7–7.4） | 7.6（4.9–10.2） | 0.026^c^ |
| Baseline characteristics |  |  |  |  |
| BMI, kg/m^2^ | 18.8±3.2 | 19.6±2.6 | 17.7±3.5 | 0.045^a^ |
| FEV_1_% predicted | 69.2（45.2–79.9） | 62.0（43.0–80.0） | 70.5（63.5–79.6） | 0.132^c^ |
| PEx | 31（68.9） | 18（72.0） | 13（65.0） | 0.614^b^ |
| PEx requiring hospitalization | 15（33.3） | 8（32.0） | 7（35.0） | 0.832^b^ |
| PA-positive results | 31（73.8） | 17（70.8） | 14（77.8） | 0.613^b^ |
| With ABPA | 13（28.9） | 6（24.0） | 7（35.0） | 0.419^b^ |
| CF-ABLE score | 2.5（2.0–4.5） | 3.5（1.0–5.0） | 2（2–3.5） | 0.635^c^ |
| 3-year prognostic score | 1.5（0–2.5） | 1.5（0–2.0） | 1.25（0.375–2.5） | 0.935^c^ |

The data are presented as No. (%), mean ± SD, or median (interquartile range). ABPA = allergic bronchopulmonary aspergillosis; BMI = body mass index; PA = *Pseudomonas aeruginosa*; PI = pancreatic exocrine insufficiency; PEx = pulmonary exacerbation; FEV_1_% = percent predicted forced expiratory volume in one second.

* There were three patients with homozygous variation of *CFTR* G970D, of whom two were **≥** 18 years and one was < 18 years at diagnosis.

^a^ Student’s t test.

^b^ Chi-square test.

^c^ Wilcoxon rank-sum test.

**e-Table 5 Univariate linear regression of characteristics related to lung function change**

| **Characteristics** | **Estimation (SE)** | **95% CI** | ***P* value** |
| --- | --- | --- | --- |
| CF-ABLE score (≥5 or 0-4.5) | 0.22 | -0.32 to 0.76 | 0.43 |
| 3-year prognostic score (≥4 or 0-3.5) | 0.18 | -0.62 to 0.98 | 0.67 |
| G970D (with or without) | 0.66 | -1.55 to 2.87 | 0.56 |
| PA (positive or negative) | -0.42 | -3.08 to 2.25 | 0.76 |
| *MRSA* (positive or negative) | -0.38 | -3.64 to 2.88 | 0.82 |
| *Mycobacterium abscessus* (positive or negative) | -0.28 | -3.87 to 3.32 | 0.88 |
| *Burkholderia cepacia* (positive or negative) | -1.14 | -8.04 to 5.75 | 0.75 |
| ABPA (yes or no) | 0.63 | -1.71 to 2.96 | 0.6 |
| Sudan III (positive or negative) | -1.34 | -3.66 to 0.98 | 0.27 |
| PEx requiring hospitalization (yes or no) | -1.85 | -4.07 to 0.36 | 0.11 |
| BMI | -0.16 | -0.51 to 0.18 | 0.36 |

ABPA = allergic bronchopulmonary aspergillosis; BMI = body mass index; CI = confidence interval; MRSA = methicillin-resistant *Staphylococcus aureus*; PA = *Pseudomonas aeruginosa*; PEx = pulmonary exacerbations.


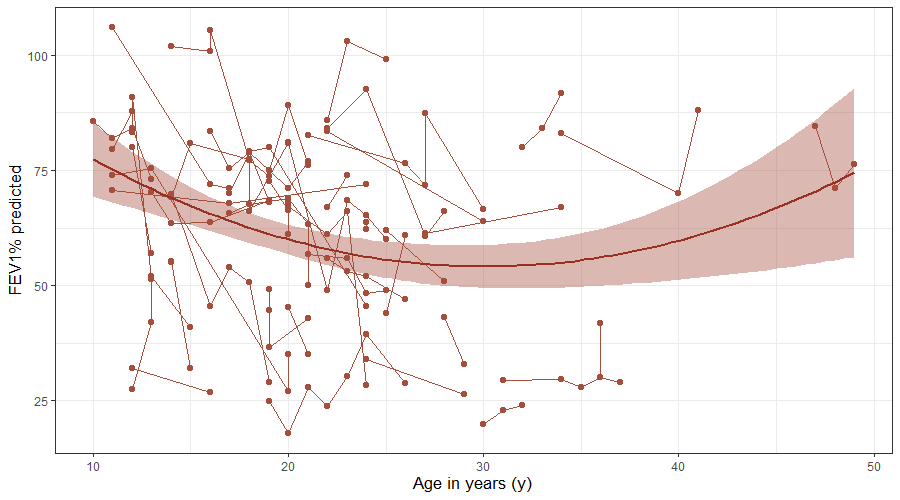


e-Figure 1 -*Graph showing the overall predicted trajectory of FEV_1_% predicted from mixed-effects model (red line), by age, and actual observed individual trajectories of each cystic fibrosis patient in the study (red lines with dots).*


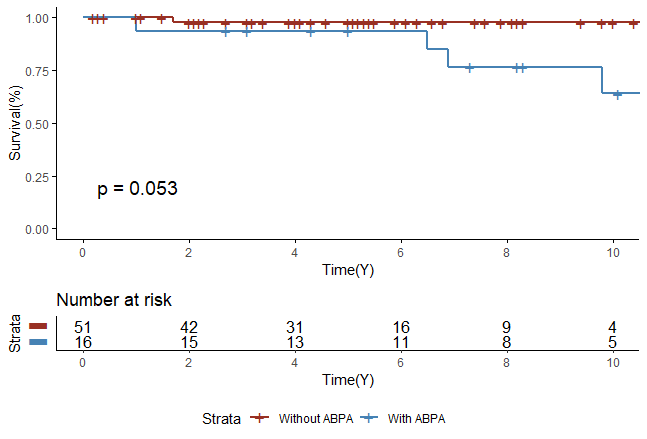


e-Figure 2 –*Kaplan-Meier survival curves for patients with cystic fibrosis by with ABPA or not.* *ABPA = allergic bronchopulmonary aspergillosis.*
